# The Association of Computed Tomography-Assessed Body Composition with Mortality in Patients with Necrotizing Pancreatitis

**DOI:** 10.1007/s11605-016-3352-3

**Published:** 2017-03-15

**Authors:** Janneke van Grinsven, Jeroen L. A. van Vugt, Arvind Gharbharan, Thomas L. Bollen, Marc G. Besselink, Hjalmar C. van Santvoort, Casper H. J. van Eijck, Djamila Boerma, for the Dutch Pancreatitis Study Group

**Affiliations:** 1 Department of Surgery, Academic Medical Center, Amsterdam, The Netherlands; 2 St Antonius Hospital, Nieuwegein, The Netherlands; 3 Department of Surgery, St Antonius Hospital, Nieuwegein, The Netherlands; 4 Department of Surgery, Erasmus MC University Medical Center, Rotterdam, The Netherlands; 5 Department of Radiology, St Antonius Hospital, Nieuwegein, The Netherlands

**Keywords:** Necrotizing pancreatitis, Body composition, Skeletal muscle mass, Skeletal muscle density, Visceral adipose tissue

## Abstract

**Background:**

Identification of patients with necrotizing pancreatitis at high risk for a complicated course could facilitate clinical decision-making. In multiple diseases, several parameters of body composition are associated with impaired outcome, but studies in necrotizing pancreatitis are lacking.

**Methods:**

A post hoc analysis was performed in a national prospective cohort of 639 patients with necrotizing pancreatitis. Skeletal muscle mass, skeletal muscle density, and visceral adipose tissue were measured at the third lumbar vertebra level (L3) on contrast-enhanced computed tomography (CT) within 10 days after initial admission and 1 month thereafter.

**Results:**

In total, 496 of 639 patients (78%) were included. Overall mortality rate was 14.5%. Skeletal muscle mass and density and visceral adipose tissue on first CT were not independently associated with in-hospital mortality. However, low skeletal muscle density was independently associated with increased mortality in patients ≥65 years (OR 2.54 (95%CI 1.12–5.84, *P* = 0.028). Skeletal muscle mass and density significantly decreased within 1 month, for both males and females, with a median relative loss of muscle mass of 12.9 and 10.2% (both *P* < 0.001), respectively. Skeletal muscle density decreased with 7.2 and 7.5% (both *P* < 0.001) for males and females, respectively. A skeletal muscle density decrease of ≥10% in 1 month was independently associated with in-hospital mortality: OR 5.87 (95%CI 2.09–16.50, *P* = 0.001).

**Conclusion:**

First CT-assessed body composition parameters do not correlate with in-hospital mortality in patients with necrotizing pancreatitis. Loss of skeletal muscle density ≥10% within the first month after initial admission, however, is significantly associated with increased mortality in these patients.

## Introduction

Acute pancreatitis is the most common reason for acute gastrointestinal hospital admission.^1^ Necrotizing pancreatitis develops in around 20% of patients.^2^^,^ 3 Depending on the presence of organ failure, necrotizing pancreatitis is classified as moderate severe or severe pancreatitis.^4^ Mortality increases, and severe morbidity rates exceed 40%, particularly when the necrosis is infected.^5^ Infected necrosis occurs in around 30% of patients with necrotizing pancreatitis and is in general an indication for invasive intervention (i.e., catheter drainage, if necessary followed by a necrosectomy).^6^^,^ 7 Necrotizing pancreatitis is characterized by a variable clinical course. To further improve outcome of patients with necrotizing pancreatitis, identification of determinants associated with high risk of mortality is needed in these patients. Several scoring systems, both radiological and clinical, have been developed to predict the severity of acute pancreatitis at hospital admission with comparable moderate accuracy for predicting mortality.^8^ All radiologic scoring systems focus on pancreatitis-associated findings (such as necrosis, pancreatic collections, and inflammatory changes), but factors associated with body composition are not part of any of these systems.

Body composition parameters that can easily and reliably be assessed on computed tomography (CT), such as skeletal muscle mass and skeletal muscle density (i.e., a measure for skeletal muscle quality and intramuscular fat infiltration), are predictive factors for poor outcome in various populations, particularly within surgical oncology,^9^^,^ 10 but also in liver transplant, vascular surgery, intensive care, and trauma patients.^11^^–^ 14 Furthermore, the loss of skeletal muscle mass, for example during chemotherapy, is associated with poor outcome.^15^ Visceral adipose tissue, the metabolically active component of total body adipose tissue, is another body composition measure that can be assessed on CT. Visceral obesity is associated with impaired outcome after surgery for various malignancies, such as colorectal, adrenorenal, and hepatocellular carcinoma.^16^^–^ 18 Moreover, a recent study suggests that android fat distribution may predict the severity of acute pancreatitis.^19^

Therefore, the aim of this study was to investigate the association between parameters of body composition (i.e., skeletal muscle mass, skeletal muscle density, and visceral adipose tissue) at onset of disease with in-hospital mortality in patients with necrotizing pancreatitis and to analyze whether skeletal muscle loss during the course of disease is associated with mortality in these patients.

## Methods

### Patients

A post hoc analysis of a prospective, observational cohort consisting of 639 necrotizing pancreatitis patients was performed. This cohort was collected in 21 Dutch hospitals (eight Dutch university medical canters and 13 teaching hospitals) from 2004 to 2008.^3^ All patients who had an abdominal CT examination performed of sufficient quality (i.e., complete images, no artifacts, and contrast-enhanced) within the first 10 days after initial admission were included. Patients with unknown body height were excluded from analyses including skeletal muscle mass, because skeletal muscle mass is expressed in the L3 muscle index (cm^2^/m^2^).^23^ Furthermore, CTs performed 30 days (±15 days) thereafter were also collected (i.e., 1-month CT). Skeletal muscle depletion was defined as a decrease exceeding 10% percent, as this was considered clinically relevant. Severe skeletal muscle depletion was defined as a decrease exceeding 25%.

### Skeletal Muscle and Adipose Tissue Measurements

Body composition measurements were performed on routinely performed contrast-enhanced abdominal CTs using FatSeg. This software program was developed at the Erasmus MC University Medical Center to perform cross-sectional soft tissue (i.e., skeletal muscle and adipose tissue) measurements on CT using the MeVisLab development environment for medical image processing and visualization version 2.4 (available from http://www.mevislab.de), as previously described.^20^ In short, the cross-sectional skeletal muscle mass area was measured with manually tracing inner and outer contours using a preset Hounsfield unit (HU) range of −30 to +150 on the level of the third lumbar vertebra (L3) on which both transversal processes were visible. The following muscles were included: psoas, paraspinal, transverse abdominal, external oblique, internal oblique, and rectus abdominis. The cross-sectional skeletal muscle area was corrected for height squared (m^2^), resulting in the L3 muscle index (cm^2^/m^2^).^21^ Mean skeletal muscle attenuation (in HU) was used as a measure of skeletal muscle density, with low skeletal muscle density reflecting high intramuscular adipose tissue infiltration and poor skeletal muscle quality.^22^^,^ 23 Visceral adipose tissue measurements were performed on the same slice with a preset HU range of −190 to −30. Selected intraluminal bowel content expressing the same radio density as adipose tissue was manually erased. Sex-specific tertiles for the body composition parameters were created. Data of our research group showed high inter- and intra-observer agreement for skeletal muscle and adipose tissue measurements on CT.^24^

### Baseline Characteristics

Baseline characteristics collected at admission at onset of disease were age, sex, body mass index (BMI, kg/m^2^), disease etiology, American Society of Anesthesiologists (ASA) classification, and the presence of organ failure at admission. Furthermore, the following clinical scores to predict the severity of pancreatitis were calculated: Acute Physiologic and Chronic Health Evaluation (APACHE) II score and Modified Glasgow score (i.e., Imrie score), with parameters collected within 24 and 48 h after admission, respectively. Additionally, the highest C-reactive protein (CRP) level in the first 48 h of admission was collected. APACHE II score (≥8), Modified Glasgow score (≥3), and CRP levels (≥150 mg/L) are previously described predictors for disease severity in acute pancreatitis.^25^^–^ 28 Necrotizing pancreatitis was defined according to the 2012 revised Atlanta classification.^4^ The presence of necrotizing pancreatitis was assessed on all baseline CTs by an experienced abdominal radiologist (TLB).

### Study Outcomes

The primary outcome was mortality during initial admission. Furthermore, the changes in skeletal muscle mass and skeletal muscle density between the baseline CT and CT after 1 month were calculated. Since there is great inter-slice variability in visceral adipose tissue due to the position of visceral organs and the potential development of (peri)pancreatic necrosis over time, this measurement was only performed on baseline CTs at onset of disease.

### Statistical Analysis

Data were analyzed using IBM SPSS Statistics for Windows, version 22.0 (IBM Corp., Armonk, NY, USA). Outcomes were reported as absolute numbers and percentages for categorical variables. Continuous variables were summarized as either means with corresponding standard deviations (SD) or interquartile ranges (IQR) depending on normality of distribution. The chi-square test was used to compare categorical variables. *T* tests were used for normally distributed continuous variables and the Mann–Whitney *U* test for non-normally distributed continuous variables. The change in skeletal muscle mass was assessed using the non-parametric paired Wilcoxon test. Univariable logistic regression analysis was performed to identify parameters that were associated with the outcome measures. Parameters that were associated in univariable analysis (*P* < 0.100) were entered into a multivariable logistics regressions analysis (backward stepwise elimination method). A two-sided *P* value below 0.05 was considered statistically significant for all statistical tests. First, the association between the body composition measures and in-hospital mortality was evaluated, followed by the association between the loss in skeletal muscle mass and density and mortality. Parameters representing disease severity (e.g., APACHE II and Modified Glasgow score) were excluded from this analysis, because these were assessed within the first 48 h of admission. Receiver Operation Characteristic (ROC) curves with corresponding areas under the curve (AUC) were created to test the predictive accuracy of known predictive scores (i.e., APACHE II score, Modified Glasgow score, and highest CRP level in the first 48 h of admission) and the CT-assessed body composition parameters. An AUC of 0.91–1.00 was considered excellent, 0.81–0.90 good, 0.71–0.80 fair, 0.61–0.70 poor, and 0.51–0.60 very poor.

## Results

### Patients and Baseline Characteristics

**Fig. 1:**
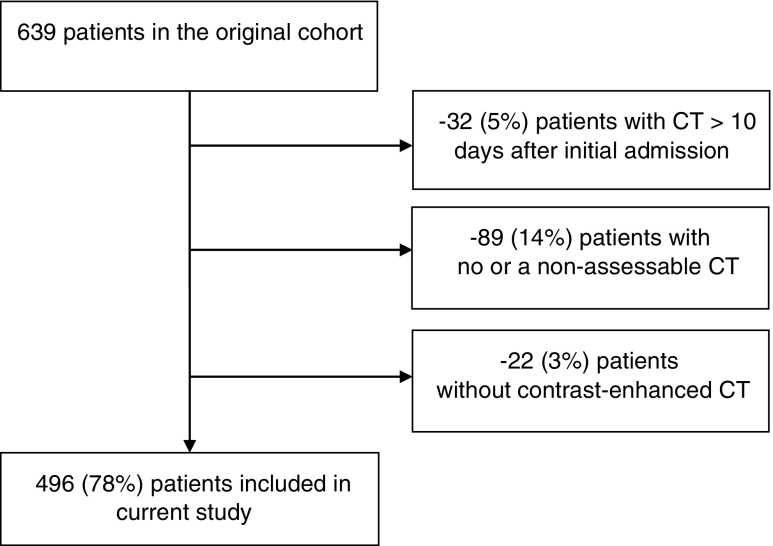
Inclusion flowchart

Of 639 patients, 143 patients (22%) were excluded either because the baseline CT was performed later than 10 days after initial admission, no sufficient (assessable) CT was available, or no contrast-enhanced CT was performed (Fig. 1). The final study cohort consisted of 496 patients (62% males) with a median age of 58 (IQR 45–70) and BMI of 26.7 (IQR 25.0–30.2) kg/m^2^. Baseline characteristics of the included patients are shown in Table 1. Baseline characteristics and study outcome (in-hospital mortality) did not significantly differ between included and excluded patients, except the presence of pancreatic necrosis (included patients 48.4% versus excluded patients 58.7%, *P* = 0.029) and BMI (included patients median BMI 26.7 kg/m^2^ versus excluded patients median BMI 28.9 kg/m^2^, *P* = 0.044). Body height was unknown for 94 (19.0%) patients.

**Table 1:** Baseline characteristics of 496 patients with necrotizing pancreatitis

Characteristic	All patients (*n* = 496)
Age, years (IQR)	58 (45–70)
Males (%)	308 (62)
BMI, kg/m^2^ (IQR)^a^	26.7 (25.0–30.2)
<18.5 (%)	3 (1)
18.5–24.9 (%)	61 (25)
25–29.9 (%)	120 (48)
≥30 (%)	64 (26)
Etiology (%)	
Biliary	240 (48)
Alcohol	114 (23)
Other	51 (10)
Unknown	91 (18)
ASA classification on admission (%)	
I (healthy status)	149 (30)
II (mild systemic disease)	272 (55)
III (severe systemic disease)	75 (15)
Organ failure at admission (%)	52 (11)
Predicted severity of pancreatitis	
APACHE II score on admission (IQR)	8 (5–11)
APACHE II score ≥8 on admission (%)	254 (51)
Modified Glasgow score within first 48 h of admission (IQR)	3 (2–5)
Modified Glasgow score ≥3 (%)	320 (65)
Highest CRP level in first 48 h of admission, mg/L (IQR)	291 (213–381)
CRP level ≥150 mg/L (%)^b^	397 (86)
Pancreatic necrosis (%)	240 (48)
Time between admission and 1-month CT, days (IQR)	2 (0–5)

Continuous variables are provided as mean (±Standard Deviation [SD]) or median (interquartile range [IQR]) depending on normality of distribution

*BMI* body mass index, *ASA* American Society of Anesthesiologists, *APACHE-II* Acute Physiologic and Chronic Health Evaluation (APACHE)-II, *CRP* C-reactive protein, *CT* computed tomography

^a^Available for 248 patients

^b^Available for 463 patients

### Body Composition Measurements

For males, the median L3 muscle index and muscle density were 53.7 (IQR 47.5–59.4) cm^2^/m^2^ and 34 (28–40) HU, respectively, and for females 44.6 (IQR 38.6–50.2) cm^2^/m^2^ and 28 (IQR 20–35) HU, respectively. The median visceral adipose tissue area was 234.3 (IQR 172.2–308.1) cm^2^ for males and 156.8 (IQR 104.1–220.0) cm^2^ for females.

### Association of Body Composition with Mortality

The median time interval between initial admission and the first abdominal CT was 2 days (IQR 0–5). The mortality rate was 14.5% (72/496) and did not significantly differ between included and excluded (14.7%) patients (*P* = 0.960). A non-significant association between the lowest L3 muscle index tertile and mortality was found (OR 0.976 (95%CI 0.507–1.878), *P* = 0.942). Significant univariable associations between the lowest HU tertile and highest VAT tertile and mortality were found. Nevertheless, the lowest HU tertile and the highest VAT tertile were not independently associated with mortality in multivariable analysis after correcting for age, ASA classification, the presence of pancreatic necrosis, and the presence of organ failure at admission: adjusted OR 1.132 (95%CI 0.617–2.078), *P* = 0.688 and 1.311 (95%CI 0.732–2.349), *P* = 0.363 (Table 2).

**Table 2:** Univariable and multivariable logistic regression analysis for risk factors for in-hospital mortality (*n* = 496, 72 patients deceased)

	Univariable OR (95% CI)	*P*	Multivariable OR (95% CI)	*P*
Sex (male)	0.78 (0.47–1.29)	0.331		
Age (years)	1.04 (1.02–1.06)	<0.001	1.04 (1.02–1.06)	<0.001
BMI (kg/m^2^)^a^				
20.0–24.9 (normal)	1 (ref)			
<18.5 vs normal	2.55 (0.21–30.89)			
>25.0–29.9 vs normal	0.62 (0.26–1.51)			
≥30 vs normal	0.73 (0.27–1.99)	0.553		
ASA I–II	1 (ref)			
ASA III	4.33 (2.46–7.62)	<0.001	4.026 (2.17–7.49)	<0.001
Organ failure at admission	5.22 (2.79–9.76)	<0.001	5.49 (2.74–10.99)	<0.001
Pancreatic necrosis	2.42 (1.43–4.09)	0.001	2.98 (1.65–5.38)	<0.001
L3 muscle index (cm^2^/m^2^)				
Highest and mid tertile	1 (ref)			
Lowest tertile	0.98 (0.51–1.88)	0.942		
Skeletal muscle density (HU)				
Highest and mid tertile	1 (ref)			
Lowest tertile	2.37 (1.43–3.92)	0.001	1.13 (0.62–2.08)	0.688
VAT (cm^2^)				
Highest and mid tertile	1 (ref)			
Lowest tertile	1.88 (1.130–3.13)	0.015	1.31 (0.73–2.35)	0.363

*BMI* body mass index, *ASA* American Society of Anesthesiologists, *VAT* visceral adipose tissue area, *OR* odds ratio, *CI* confidence interval

^a^Available for 248 patients

As skeletal muscle mass and density are strongly associated with age,^29^ subgroup analyses were performed in patients aged <65 and ≥65 years. Patients aged ≥65 in the lowest skeletal muscle density tertile showed an increase mortality rate (29.3%) compared with patients in the mid and highest tertile (13.5%), *P* = 0.008. Skeletal muscle density in the lowest tertile was associated with an increased risk of in-hospital mortality (adjusted OR 2.54 (95%CI 1.12–5.84), *P* = 0.025) in patients ≥65 years, independently of ASA classification, organ failure at admission, and pancreatic necrosis. An incremental increase in skeletal muscle density showed a protective effect on mortality (adjusted OR 0.94 (95%CI 0.90–0.99), *P* = 0.010).

### Predictive Accuracy of Established Risk Parameters and Body Composition Parameter

**Fig. 2:**
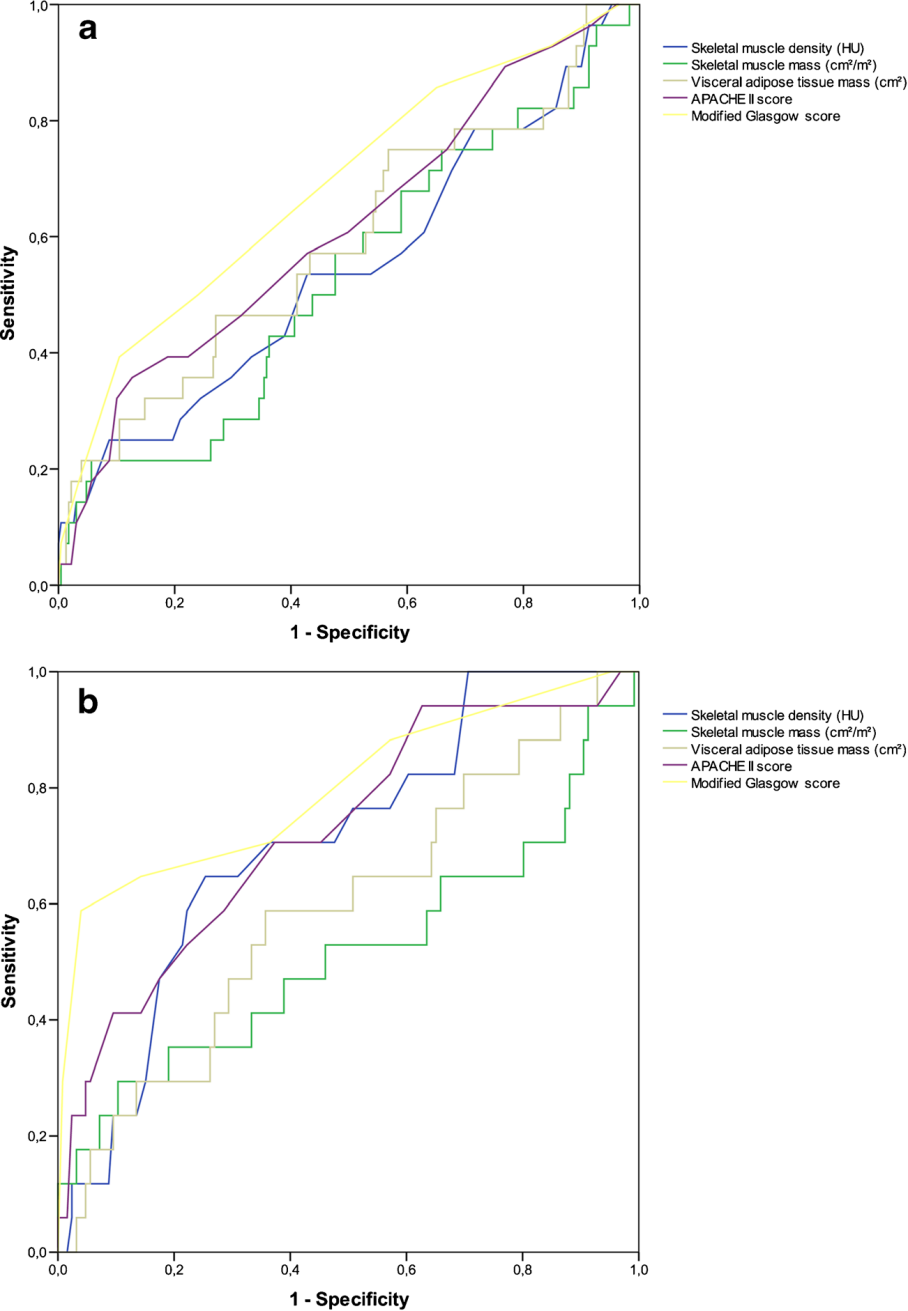
Receiver Operating Curves (ROC) for severity scores (i.e., APACHE II and Modified Glasgow scores) and CT-assessed body composition parameters. Skeletal muscle density (HU) and mass (cm^2^/m^2^) values have been inverted because lower values represent higher risk. **a** Males. **b** Females

The ROC curves are depicted in Fig. 2, for males and females, separately. In males (Fig. 2a), APACHE II and Modified Glasgow scores showed a poor predictive value (AUC 0.62 and 0.62, respectively) and all body composition parameters a very poor predictive value (AUC ranging from 0.53 to 0.59). In females (Fig. 2b), APACHE II and Modified Glasgow score showed a fair predictive value with AUCs of 0.72 and 0.80, respectively. Skeletal muscle and visceral adipose tissue mass showed a very poor predictive value (AUC 0.52 and 0.59, respectively), whereas skeletal muscle density showed a fair predictive value with an AUC of 0.71.

### Skeletal Muscle Mass and Density Loss During the Disease Course

In total, 189 patients (66.7% male, median age 58 years) had a baseline CT and a CT 1 month thereafter with a median time interval of 29 (IQR 25–33) days. In-hospital mortality in these patients was 14.3% (27/189). For males, the median skeletal muscle mass (i.e., cross-sectional skeletal muscle area) decreased from 169.7 (IQR 153.2–193.9) cm^2^ to 147.9 (IQR 131.5–166.7) cm^2^ and for females from 125.2 (IQR 112.1–137.3) cm^2^ to 111.9 (98.9–121.6) cm^2^ (both *P* < 0.001). This resulted in a median relative difference of 12.9% (IQR 5.8–20.9) and 10.2% (IQR 1.4–18.8), respectively. For males, the median skeletal muscle density decreased from 33 (IQR 27–39) HU to 31 (IQR 23–38) HU and for females from 27 (IQR 20–33) HU to 23 (IQR 15–35) HU (both *P* < 0.001), with median relative differences of 7.2% (IQR −9.1–22.7) for males and 7.5% (IQR −15.0–32.3) for females. Corrected for the time interval between the baseline CT and the follow-up CT, significant decreases in cross-sectional skeletal muscle area of 0.43% (IQR 0.15–0.72) and in skeletal muscle density of 0.28% (IQR −0.41–0.97) per day were observed (*P* < 0.001 and *P* = 0.035, respectively).

In total, 110 (58.2%) patients lost ≥10% of cross-sectional skeletal muscle area. Eighty-nine (47.1%) patients experienced ≥10% decrease in skeletal muscle density. Mortality rate was not significantly different between patients with compared to patients without ≥10% skeletal muscle mass loss (14.5 versus 13.9%, *P* = 0.904), whereas patients with ≥10% decrease in skeletal muscle density showed an increased risk of mortality (24.7 versus 5.0%, *P* < 0.001). More than 10% skeletal muscle density loss (adjusted OR 5.87 (95%CI 2.09–16.50), *P* = 0.001) was an independent risk factor for in-hospital mortality, after correcting for sex and age (Table 3). These patients had a significantly longer hospital (median 70 (IQR 45–106) versus 45 (IQR 23–71) days, *P* < 0.001) and intensive care unit (median 0 (IQR 0–11) versus 14 (IQR 2–38) days, *P* < 0.001) stay compared with patients <10% skeletal muscle density loss. Twenty-eight (14.8%) and 48 (25.4%) patients experienced decreases in skeletal muscle mass and density that exceeded 25%. More than 25% skeletal muscle mass loss was not independently associated with increased in-hospital mortality (adjusted OR 2.72 (95%CI 0.98–7.56), *P* = 0.055) after correcting for age, while a decrease of ≥25% skeletal muscle density was associated with increased in-hospital mortality (adjusted OR 5.4 (95%CI 2.25–13.04), *P* < 0.001).

**Table 3:** Association between skeletal muscle mass and skeletal muscle density loss within 1 month and mortality (*n* = 189, 27 patients deceased)

	Univariable OR (95% CI)	*P*	Multivariable OR (95% CI)	*P*
Sex (male)	0.688 (0.30–1.59)	0.380	0.82 (0.33–2.04)	0.669
Age (years)	1.045 (1.01–1.08)	0.007	1.04 (1.01–1.08)	0.016
≥10% Skeletal muscle mass loss (cm^2^)	1.05 (0.46–2.41)	0.904	0.75 (0.29–1.93)	0.556
≥10% Skeletal muscle density loss (HU)	6.24 (2.25–17.30)	<0.001	5.87 (2.09–16.50)	0.001

*OR* odds ratio, *CI* confidence interval

## Discussion

This is the first, multicenter study to analyze the association between CT-assessed body composition parameters and mortality in patients with necrotizing pancreatitis. None of the early body composition parameters chosen was associated with in-hospital mortality overall. Nevertheless, low skeletal muscle density was independently associated with mortality in patients aged ≥65. Furthermore, our data suggest that skeletal muscle depletion of ≥10% after 1 month in patients with necrotizing pancreatitis may identify patients at risk for mortality.

We hypothesized that CT-assessed skeletal muscle mass corrected for body height (low L3 muscle index) was associated with impaired outcome in patients with necrotizing pancreatitis. Although the association between body composition (i.e., skeletal muscle and visceral adipose tissue mass) and outcome has been investigated extensively in cancer populations,^9^^,^ 10 some studies have shown its association in benign diseases.^11^^–^ 14 Low skeletal muscle density was shown to be of prognostic value in patients with renal cell carcinoma,^30^ lymphoma,^31^ melanoma,^32^ and various other malignancies.^22^ Moreover, a previous study reported lower mean values of the cross-sectional muscle area, as well as a lower mean BMI in patients with chronic pancreatitis compared with the current study cohort.^33^ In the current study among patients with necrotizing pancreatitis, however, neither skeletal muscle mass nor density was related to mortality in the overall population. There are two possible explanations for the differing findings. First, the acute onset of the disease in these a priori relatively healthy patients (85% ASA classification 1–2) could explain the higher index values in our cohort as there was no underlying disease or catabolic state before. Previous studies on this topic primarily investigated patients with metabolically active diseases (e.g., cancer patients) or those with chronic illnesses (e.g., patients awaiting liver transplantation for cirrhosis, patients with chronic pancreatitis). Second, higher age is a significant determinant for sarcopenia (i.e., the involuntary loss of skeletal muscle mass and strength). Prior studies predominantly included older patients (e.g., patients with cancer or abdominal aortic aneurysm), whereas the age of patients with necrotizing pancreatitis is usually considerably lower (median age of 58 years in our study). Our finding in older patients is in line with a previous study on short-term outcomes in colorectal cancer patients. Especially in patients ≥65 years of age, an association was found with increased infectious complication rates, inpatient rehabilitation care, and consequently prolonged hospital stay.^29^

Besides skeletal muscle mass and density, the amount of visceral adipose tissue was also not associated with mortality in patients with necrotizing pancreatitis in the current study. This is in line with a previous study, which found no relationship between adipose tissue distribution and pancreatitis severity.^34^ In this particular study, obese patients showed worse predicted severity scores (e.g., by the Ranson score), but abdominal fat distribution was not independently associated with the actual acute pancreatitis severity and mortality. Another recent study found no difference in clinical outcome in ICU-admitted acute pancreatitis patients with or without a decrease in visceral adipose tissue mass.^35^ Future studies should further address whether routine assessment of these body composition parameters could be of added value in current (CT) severity index models.

Although necrotizing pancreatitis is innately a benign disease, it is characterized by a striking hypercatabolic metabolic state. Based on results of this study, this leads to considerable skeletal muscle loss within 1 month in some patients, which is comparable with or even larger than the loss in palliative cancer patients in 3 to 6 months.^36^ Previous studies showed that both prolonged hospital stay and inflammatory processes (such as acute pancreatitis) are known to be associated with skeletal muscle depletion.^37^^,^ 38 Also, a significant decrease in skeletal muscle mass has previously been described in various cancer patients undergoing (neo)adjuvant chemotherapy or chemo-radiation therapy. This was associated with increased incidence of postoperative complications in esophageal cancer patients.^37^ Furthermore, a decreased survival has been reported in colorectal cancer patients who experienced skeletal muscle depletion,^39^^,^ 40 but not in esophagogastric cancer patients.^41^^,^ 42 Interestingly, in patients with necrotizing pancreatitis, we found that substantial loss of skeletal muscle density (7.2 and 7.5% for males and females, respectively) in the first month after initial admission significantly correlated with mortality. The differing and positive effect of the decline of skeletal muscle mass on mortality in necrotizing pancreatitis compared with other diseases likely relates to the intensity and degree of its loss within a shorter period of time. As such, considerable loss of skeletal muscle mass and density during the course of acute pancreatitis correlates with disease severity and can identify those at high risk of mortality. Furthermore, it is conceivable that considerable alterations in body composition may prolong the period of convalescence in those who survive. A previous study among acute pancreatitis patients who were admitted at the ICU found no skeletal muscle mass loss, whereas a significant decrease in visceral adipose tissue mass was observed.^35^ However, this study cohort consisted of only 21 patients and the time interval between CTs was not described. Furthermore, inter-slice variability in visceral adipose tissue due to the position of visceral organs and the potential development of (peri)pancreatic necrosis over time may have contributed to the significant detected decrease in visceral adipose tissue mass, whereas skeletal muscle mass has been measured more reliably.

Our findings support the potential beneficial effects of nutritional support in acute pancreatitis. In the early phase, nutrition is essential as it maintains gut function, prevents ileus, and reduces bacterial overgrowth.^43^ Both in the early and late phases of the disease, active nutrient repletion helps tissue repair and healing and may potentially minimize alterations in body composition. However, nutritional support only, as any other monotherapy, will probably not be sufficient.^44^ Therefore, future research should focus on nutritional support and other pharmacological agents to counterbalance the detrimental effects of skeletal muscle depletion in patients with necrotizing pancreatitis.^44^ Alternative treatment options, such as myostatin inhibition, which reduces skeletal muscle wasting,^45^ are currently being investigated in phase II clinical trials in cancer patients^46^ and might prove useful in acute pancreatitis. Future studies on this topic are highly warranted.

This study has some limitations. First, patients were divided in sex-specific tertiles rather than using dichotomous cut-off values, since commonly used cut-off values to classify patients as (non)sarcopenic have been derived only in cancer patients.^20^^–^ 22 Using optimal stratification to find cut-off values, as has been performed in previous studies,^20^^,^ 21 was not possible due to the dichotomous character of our primary study end-point (i.e., in-hospital mortality). Another limitation is that our results apply only to patients who eventually develop necrotizing pancreatitis. We feel, however, that inclusion of patients with interstitial disease would not have altered our results in a meaningful way, as mortality is rare in patients with interstitial pancreatitis, who in general recover fast and uneventfully. Finally, as described previously, CTs were not performed routinely or at regular time intervals, but only in patients who showed predicted severe pancreatitis, deteriorated or did not improve clinically.^3^ This explains the short median time interval of 2 days between admission and the first CT. Furthermore, the comparable mortality rates between the entire cohort (14.5%) and the cohort of patients who had a CT after 1 month (14.3%) suggest that patients who had no CT after 1 month either had deceased or experienced an uneventful recovery and that early body composition was not associated with mortality. Consequently, this may have led to selection bias and results should therefore be interpreted with caution.

In conclusion, neither early skeletal muscle mass and density nor visceral adipose tissue mass are associated with in-hospital mortality in patients with necrotizing pancreatitis. However, low skeletal muscle density was independently associated with in-hospital mortality in patients ≥65 years. Furthermore, significant decreases in skeletal muscle mass and density were observed in the first month after initial admission. A decrease of ≥10% in skeletal muscle density within the first month after admission may identify patients at increased risk for mortality. Future prospective studies should investigate the true value of CT-assessed skeletal muscle mass and density, visceral adipose tissue, and its losses, to predict patient outcome in acute (necrotizing) pancreatitis and determine whether (prophylactic) treatment allows counteracting the deleterious effects of body composition alterations.
